# A case report of lithium-induced bradycardia and syncope

**DOI:** 10.1192/j.eurpsy.2025.1835

**Published:** 2025-08-26

**Authors:** M. E. Kilinc, E. Mutlu, B. Demir

**Affiliations:** 1 Department of Psychiatry, Hacettepe University, Ankara, Türkiye

## Abstract

**Introduction:**

Lithium is a mood stabilizer, causing various dose related adverse effects. Cardiac adverse effects are seen more frequently in patients with previous cardiac diseases, overdoses, and chronic use.

**Objectives:**

We report the case of a woman treated with lithium who developed bradycardia and syncope, which are rare adverse effects of lithium.

**Methods:**

A 39-year-old woman was hospitalized due to suicidal ideation during a psychotic depressive episode. She had a previous supraventricular tachycardia episode requiring ablation but was asymptomatic for a long time. On admission, she was using sertraline 100 mg/day and olanzapine 10 mg/day. Lithium was initiated at 600 mg/day due to previous suicide attempts and being in the fourth depressive episode. On the second day of lithium therapy, the patient had a syncope lasting a minute. Electrocardiogram (ECG) showed sinus bradycardia at 46 bpm and 417 msec of QTc. She was normotensive. Her serum electrolytes, myocardial enzymes, thyroid hormones, and thyroid-stimulating hormone levels were within normal limits. Lithium was stopped. The following ECG showed sinus rhythm at 65 bpm. Cardiology consultation resulted in no contraindication to lithium therapy. Lithium was reinitiated with 300 mg, no syncope or bradycardia was observed, and the patient was discharged after 9 days. Lithium concentration was 0.66 mEq/L. The Naranjo Advers Drug Reaction Scale was scored as 5, indicating a probable relationship between syncope, bradycardia, and lithum. She is still using lithium 300 mg/day for four months with no adverse-effects.

**Results:**

At both therapeutic and toxic lithium levels, ECG changes such as T-wave inversions, sinus bradycardia, sinoatrial blocks, PR prolongation, QTc prolongation, and ventricular tachyarrhythmias can be observed. Lithium cardiac adverse effects have been shown to increase with age and duration of treatment and can be seen in both therapeutic and toxic concentrations. Our case is unique, occurring in a relatively young patient in the early phase of lithium treatment.

**Conclusions:**

Other causes of bradycardia should be eliminated by performing a workup that includes calcium level, thyroid function, and cardiac workup, and checking medication interactions. Lithium-induced bradycardia is reversible upon discontinuation of lithium, but irreversible sinus node may occur when long-term lithium therapy is required. As lithium is an indispensible option for such patients with severe recurrent depression, lithium may be rechallenged with a lower dose and close followup of the ECG despite previous episodes of bradycardia and syncope.

**Disclosure of Interest:**

None Declared

